# Nutrition and its Relation to Mealtime Preparation, Eating, Fatigue and Mood Among Stroke Survivors After Discharge from Hospital - A Pilot Study

**DOI:** 10.2174/1874434600802010015

**Published:** 2008-01-28

**Authors:** Albert Westergren

**Affiliations:** Research and Development Unit, Central Hospital Kristianstad, Northeast Skåne Health Care District; The Department of Health Sciences, Kristianstad University, Kristianstad, Sweden

**Keywords:** Fatigue, mood, mealtime preparation, eating, nutrition, stroke.

## Abstract

Eating difficulties and nutritional deficits are common among persons with acute stroke and during rehabilitation. Little is known about such problems after discharge from hospital. In addition the relationship between fatigue and nutritional status among stroke survivors living in the community remains to be explored. The aim of this pilot study was to describe mealtime preparation, eating, fatigue, mood and nutritional status among persons with stroke six months after discharge from hospital and to explore associations between these factors. Patients were interviewed six months poststroke. Standardised questions and methods were used. The mean age of the 89 respondents was 77.2 (SD 6.6) years, 44 were women and 45 men. Difficulties with swallowing, ingestion and energy to eat occurred among 27%, 20% and 7% respectively. Difficulties with cooking and buying food occurred among 57% and 56% respectively and 41% were at nutritional risk. Feeling full of energy less than some of the time was experienced by 61% while 15% had felt gloomy and sad at least some of the time during the previous four weeks. Considering activities of daily living (ADL), having a less favourable nutritional status was significantly predicted by difficulties with buying food, difficulties with ingestion and being a woman. Considering psychological state (mood and energy), having a less favourable nutritional status was significantly predicted by a lack of energy and high age. This study supports the occurrence of a nutritionally related fatigue by means of “lack of energy”. The associations between poor nutritional status and fatigue can work in both directions. Thus persons with fatigue are more prone to have poor nutritional status and those with poor nutritional status are at greater risk of fatigue. Besides fatigue also difficulties with buying food and ingestion are associated with nutritional risk. As nutritional deficits occur a long time after stroke onset it is important to assess aspects of mealtime preparation and the eating process and when necessary provide food delivery service and eating assistance in order to prevent a vicious circle of undernourishment and fatigue to develop.

## INTRODUCTION

Eating difficulties and nutritional deficits are common among persons with acute stroke and during rehabilitation [1, 2]. However, little is known about such problems after discharge from hospital. In addition there is a lack of knowledge about the associations between fatigue and nutritional status in this group.

Stroke has a significant impact on health due to its high incidence, prevalence and subsequent disability rate [3]. Stroke patients can experience disabilities that influence eating habits and dietary intake. Stroke can also cause problems with mealtime-related activities such as buying and cooking food, and it can affect the mealtime situation by causing eating difficulties. Various functional impairments relating to arm movement, posture, lip closure, chewing and swallowing abilities, perception, attention and sensation contribute to eating difficulties among stroke survivors [4].

Eating difficulties are common among stroke survivors living in the community and have been found to have a negative impact on quality of life, together with mood state (Hospital Anxiety and Depression Scale, HADS), social supportand age [5]. Perry and McLaren [6] found among 206 patients at six months post-stroke a high prevalence of upper limb/motor impairment (75%), dysphagia (19%), chewing difficulties (18%) and lip closure difficulties (10%)[6]. Regarding mealtime preparation in the post-stroke period, around half of the patients do not cook and around half do not shop [7]. Mealtime related activities have only been studied to a limited extent in community-living stroke survivors.

“Normal” fatigue is a state of general tiredness that is the result of overexertion and can be ameliorated by rest. “Pathologic” fatigue is a state characterised by weariness unrelated to previous exertion levels and is usually not ameliorated by rest [8]. A corresponding concept is activity intolerance, which can be defined as a state in which an individual has insufficient physiological or psychological energy to endure or complete required or desired daily activities [9]. Further, the concept of “sustained attention” also relates to fatigue and it “reflects the ability to concentrate on a given task over long and generally unbroken periods of time without a substantial decline or large fluctuations in the performance” [10, p 12]. Lack of energy is a key concept of fatigue. In a review of literature fatigue was defined as “a feeling of physical tiredness and lack of energy that was described as pathologic, abnormal, excessive, chronic, persistent or problematic” [11,p 1715].

In some studies conducted by Westergren and colleagues fatigue has been objectively assessed in relation to eating in terms of “energy to fulfil a meal” or “alertness during meals” [2, 12]. These aspects of eating correspond to the concepts of “activity intolerance” and “sustained attention” [9, 10]. Among patients in different rehabilitation settings a lack of energy to fulfil a meal was found to be associated with undernourishment [2] and it was found to have the strongest association with undernourishment of the various eating difficulties assessed, among patients with stroke [12]. These findings are important especially as lack of energy was the only eating difficulty that did not improve in patients with stroke between admission and discharge from hospital [4], thereby increasing the risk of developing undernourishment later in the course of stroke. In addition, it was found that low energy problems were more common among women than among men both on admission and at discharge from rehabilitation [4]. A lack of energy to fulfil a meal in combination with dysphagia can be a conspicuously important obstruction to eating. It was found in another study by Westergren [13] that patients with a combination of lack of energy and dysphagia had more difficulties in eating than those who had dysphagia alone. The relationship between post-stroke fatigue and nutritional status remains to be explored among persons six months post-stroke.

Only a few studies have described self-reported fatigue in a population of stroke patients. Among 167 persons at six months post-stroke fatigue has been in 69.5%. In the same study depression related to post-stroke fatigue [14]. One study of psychosocial functioning of 50 persons post-stroke reported general tiredness in a large number of respondents at three months (54%), one year (44%), two years (42%) and three years (44%) post-stroke [15]. Ingels, Eskes, & Phillips [16] found that 68% of 88 respondents at three to 13 months post-stroke reported fatigue problems, as measured by the Fatigue Impact Scale (FIS). When asked if they “felt full of energy” on a depression rating scale, 78% answered no [16]. Knowledge about determinants of post-stroke fatigue is still limited, especially with regard to the relationship between fatigue and nutritional status.

The aim of this pilot study was to describe mealtime-related problems, fatigue, mood and nutritional status among persons with stroke six months after discharge from hospital and to explore associations between these factors.

## MATERIALS AND METHODS

A cross-sectional and explorative design was used using structured interviews and assessments. The data were collected during autumn 2003 and spring 2004.

All patients discharged from a stroke unit at a hospital in southern Sweden during a period of 6 months were asked to participate in the study. No predefined criteria for exclusion was used in order to also include those with minor cognitive and/or minor communication difficulties. In total 120 persons were eligible for inclusion, of which 89 (74%) agreed to participate in the study. There were no differences between those who did not enrol in the study (n = 31) and those included (n = 89) regarding age, gender, general health, life situation right now, and worries about own health. The main reasons for dropout were lack of energy (n = 11), cognitive and communication difficulties (n = 4)[17].

The study sample has been extensively described in two previous articles [17, 18]. The results indicate that most of the patients were in need of help with home-care activities while most only had a moderate dependency in personal activities of daily living. In addition most patients rated their general health as good and no cognitive impairment was found in 49 out of the 89 included patients [17, 18]. Thus a high proportion of the patients were relatively highly functional six months after their stroke.

Interviews were carried out by the same Registered Nurse, who was experienced in stroke care, and the majority were conducted in the person’s own home (n = 71), some in special accommodation (n = 15) and a few in other places (n = 3). A questionnaire with structured questions was used for the interviews.

### Nutritional Status

Nutritional status was estimated using six questions from MNA-SF (Mini Nutritional Assessment-Short Form ®) [19] including Body Mass Index (BMI), declined food intake over the past three months due to loss of appetite/digestive problems or swallowing difficulties, weight loss during last three months, mobility, neuropsychological problems and psychological stress or acute disease in the past three months. The scores from all questions are summed together giving a minimum score of 0 and a maximum score of 14 with a high score indicating better nutritional status. If the score is 12 points or greater, the patient is not at nutritional risk and if 11 points or less, the patient may be at nutritional risk.

### Mealtime-Related Activities

Mealtime-related activities were assessed using two variables from the well-established “ADL staircase” [20]. These two variables were cooking (graded from 0 = no problem to 2 = does not cook) and buying food (graded from 0 = no problem to 2 = does not shop).

### Eating Activity

Eating was specified as ingestion (0-3), swallowing (0-3) and energy to eat (0-3). The grading of these variables, presented in brackets, ranged from no problems (the lowest value) to having the problem/severe problems (the highest value). These three variables are of relevance for eating and were formulated based on findings from a previous study [2] in which a factor analysis of nine eating activities among 520 elders in hospital rehabilitation was performed. The nine eating activities were found to belong to three components. The first one was labelled ingestion, which included manipulation of food on the plate, transport of food to the mouth and sitting position (Cronbach’s alpha (α) of 0.76). The second component was labelled swallowing, and included opening and/or closing the mouth, manipulation of food in the mouth and swallowing difficulties (α 0.73). The third component, labelled energy to eat, included alertness, unusual eating time and eating ¾ or less of served food (α 0.60). Based on this result, it was possible to define the three questions reflecting the eating ability, i.e. ingestion, swallowing and energy to eat [17].

### Fatigue and Mood

Fatigue and mood were estimated with two questions taken from one instrument. The 12-item Short Form Health Survey, SF-12, is used to measure subjective physical and mental health [21]. From that questionnaire two questions were selected for this study. First, “Have you felt full of energy during the last four weeks?” ranged from 0 (all the time) to 5 (none of the time) (SF-12 question 10). Secondly, “Have you felt gloomy and sad during the last four weeks?” ranged from 0 (none of the time) to 5 (all the time) (SF-12 question 11).

### Ethical Considerations

Information about the study was given to the potential participants when contacted by phone. If they agreed, a time for a home visit was scheduled. At the home visit, written consent was obtained from participant or a significant other. It was stressed that participation was voluntary. The Ethics Committee of the Medical Faculty, Lund University, approved the study.

### Analysis

Descriptive and correlation statistics (multivariate logistic regression analysis) were used. In the regression analyses nutritional status score, dependent variable, was dichotomised (after having reversed the score, 0 = nutritional score >12 and 1 = nutritional score < = 11 = worse). Besides age the independent variables entered as dichotomised variables in the first regression analysis were: gender (0 = male; 1 = woman), difficulties with mealtime preparation (buying and cooking food, 0 = no difficulties, 1 = having difficulties), eating difficulties including ingestion, swallowing and energy to eat (0 = no difficulties; 1 = having difficulties). Besides age the independent variables entered as dichotomised variables in the second regression analysis were: gender, feeling full of energy (0 = some to all of the time; 1 = no, a little) and feeling gloomy and sad (0 = no, a little; 1 = some to all of the time).

Model fit was measured with the Hosmer and Lemeshow goodness-of-fit test, which measures differences between actual and predicted values of the dependent variable. Good model fit is indicated by a non-significant and small chi-square value, indicating no differences in actual and predicted dependent values [22]. *p*-values below 0.05 were considered statistically significant. Analyses were performed with SPSS 13.0.

## RESULTS

The mean age of participants (n = 89) was 77.2 years and the distribution of men and women was almost equal (n = 45/44). Most were married (n = 43) or widowed (n = 32), and more were living alone (n = 49) than living together with someone else (n = 40). Most lived in their own (ordinary) homes (n = 72). The most common diagnosis was cerebral infarct (n = 77), and most were discharged to their own homes after the hospital stay (n = 71) (Table **1**).

Difficulties with swallowing, ingestion and energy to eat occurred among 27%, 20% and 7% respectively. Difficulties with cooking and buying food occurred among 57% and 56% respectively (Table **2**).

The mean nutritional score was 11.8 (SD 2.0, Median 13, IQR 11.0-13.0) and 41% were considered to be at nutritional risk.

Feeling full of energy less than some of the time was experienced by 61% while 15% had felt gloomy and sad at least some of the time during the previous four weeks (Table **3**).

Considering activities of daily living (ADL), having a less favourable nutritional status was significantly predicted by difficulties with buying food, difficulties with ingestion and being a woman (Table **4**).

Considering psychological state, having a less favourable nutritional status was significantly predicted by energy and age but not by feeling gloomy/sad and gender (Table **5**).

## DISCUSSION

Less than half experienced themselves as full of energy most of the time and 15% reported feeling gloomy and sad at least some of the time during the previous four weeks. Of the ADL variables having difficulties with buying food and ingestion predicted nutritional risk and of the psychological variables energy could predict nutritional status while feeling gloomy and/or sad could not.

Single item measures were used to indicate gloominess/sadness and fatigue in this pilot study. Reasons for this were to minimise respondent burden and to get a “snap shot” of the topics. Bowling [23,p 342] stated “while shorter instruments are more limited than longer measures, they have obvious benefits for both research and policy in terms of reduced burden and costs, and ease of interpretation”. Patrician [24] argues in a review, that nurse researchers should seriously consider single-item measures as part of their methodological research “toolkit”. Further on, the single item measure has the advantage of simplicity and can be valid and reliable however it is at expense of detail. Single item measures have been extensively used [23] and have been used among patients with stroke to capture depression and fatigue [25] and a single item measures about lack of energy has been found to be significantly correlated with multi item measures of fatigue among cancer patients [26]. Even though, studies are needed to explore the associations between fatigue and nutritional status using also multi item measures and full length Mini Nutritional Assessment [27].

Although difficulties exist in defining fatigue it is clear that there is a component of “energy” in it [11]. As seen in earlier studies, “energy” can be measured objectively during mealtimes and then reflects a situation-bound fatigue corresponding to activity intolerance and lack of ability to sustain attention [2, 9, 10, 12]. In addition, it seems as if nutritionally related fatigue can be measured by a question reflecting lack of energy over the last four weeks, as shown in this pilot study. Ingels *et al. *[16] used a similar question in their study of 88 respondents post-stroke and 78% answered that they felt a lack of energy, while the corresponding figure in this study was 61% (full of energy some, a little or none of the time) or 82% if considering any level of not feeling full of energy.

Even if this is the first study giving evidence for a relationship between nutritional status and fatigue post-stroke there are other studies using related variables that have found comparable results. For instance, in a study by Westergren *et al.* [4] a relationship was found between alertness and undernourishment. In another study it seemed that patients with dysphagia in combination with low energy were more prone to develop respiratory infections as well as undernourishment [13]. However, a relationship between fatigue and nutritional status among stroke survivors six months post-stroke has not been shown until now.

Women are especially vulnerable to undernourishment. In this study it was shown that being a woman could predict a poor nutritional status. A study by Westergren *et al.* [4] among persons in stroke rehabilitation showed that women were older, ate less on admission and at discharge than men, and improved less than men. Further, the number of low energy problems at discharge was higher for women than for men. This finding is in accordance with the findings of this study, i.e. women are at special risk of undernourishment both in hospital and after discharge.

Energy deficit and undernourishment interact in both directions. Persons with acute stroke often experience fatigue that causes difficulties with eating [4]. It might even happen that they stop eating before they have satisfied their hunger, as they need to rest or even fall asleep. If patients eat and drink too little in relation to their needs the fatigue most likely escalates. Conversely, a person with stroke might experience less motivation to eat and/or difficulties with mealtime preparation that cause them to eat and drink too little. This can cause fatigue and undernourishment. Thus, it seems important to stop a vicious circle from developing, regardless of what direction it works in. There are several measures to take in order to improve nutritional intake while less is known about effective measures to decrease fatigue.

## CONCLUSIONS

As nutritional deficits occur a long time after stroke onset it is important to assess aspects of mealtime preparation as well as the eating process and when necessary provide food delivery service and eating assistance in order to prevent a vicious circle of undernourishment and fatigue to develop. The associations between poor nutritional status and fatigue are likely to work in both directions. Thus persons with fatigue are more prone to have poor nutritional status and those with poor nutritional status are at greater risk of fatigue. Women and those with high age are especially at nutritional risk.

## Figures and Tables

**Table 1.** Demographic Data for Stroke Survivors

	Included, n = 89
**Age, mean (SD)** **Women, %**	77.2 (6.6) 49
**Marital status, %**	
Married Unmarried Widowed Divorced	49 9 37 5
**Living status, %**	
Living together with someone else Living alone	45 55
**Diagnosis, %**	
Cerebral infarct Cerebral haemorrhage Other	88 7 5
**Coming to hospital from, %**	
Own home Other	98 2
**Discharged from hospital to, %**	
Own home Special accommodation Other	81 17 2

**Table 2.** Description of Eating Difficulties and Mealtime-Related Activities Among Stroke Survivors

Eating Difficulties	n = 89
**Swallowing, %**	
Swallows without adjustment Swallows with adjustment Needs assistance and adjustment Cannot swallow	73 24 3 0
**Ingestion, %**	
No assistive devices, adjustment, assistance Eats with assistive devices/adjustment Needs assistance from one person Does not ingest	80 17 1 2
**Energy to eat, %**	
Eats whole meal, no fluctuation in perform. Eats whole meal, some fluct. in perform. Stops due to reduced strength (not satiety) Can not eat, probably due to reduced strength	93 1 4 2
**Cooking, %**	
Cooks at least 3 times a week Needs some assistance in the activity Does not perform the activity at all	43 9 48
**Shopping, %**	
Shops at least once a week Gets to store with help from one person Does not shop/needs some help in activity	44 16 40

**Table 3.** Energy and Gloominess

	n = 89
**Feeling full of energy, % **	
All the time Most of the time A great deal of the time Some of the time A little of the time None of the time	19 12 9 18 25 18
**Feeling gloomy and sad, %**	
None of the time A little of the time Some of the time A great deal of the time Most of the time All the time	57 28 11 2 1 1

**Table 4.** Eating and Mealtime Related Activities Associated with Nutritional Status (0 = Better Score, 1 = Worse Score, Logistic Regression, Forward Conditional Method) Among Stroke Survivors

	Exp (B)	95% CI for Exp (B)	p-Value
Constant	0.232		0.005
Gender (0 = woman)	0.248	0.069-0.888	0.032
Ingestion	9.995	1.607-62.189	0.014
Buying food	10.221	2.797-37.347	0.000

Hosmer and Lemeshow Test, p-value 0.098, Chi-square 9.305, Overall Percentage correct 60.3%, Cox & Snell R Square 0.348, Nagelkerke R Square 0.471. Variables not significantly associated with nutritional status: age, cooking, swallowing and energy to eat.

**Table 5.** Energy and Mood Associated with Nutritional Status (0 = Better Score, 1 = Worse Score) (Logistic Regression, Forward Conditional Method) Among Stroke Survivors

	Exp (B)	95% CI for Exp (B)	p-Value
Constant	0.000		0.005
Age	1.109	1.023-1.204	0.013
Energy	3.065	1.097-8.557	0.033

Hosmer and Lemeshow Test,p-value 0.802, Chi-square 4.572, Overall Percentage correct 72.5%, Cox & Snell R Square 0.235, Nagelkerke R Square 0.315. Variables not significantly associated with nutritional status:feeling gloomy and sad and gender.
